# Effects of Neurogenin 3 Induction on Endocrine Differentiation and Delamination in Adult Human Pancreatic Ductal Organoids

**DOI:** 10.3389/ti.2025.13422

**Published:** 2025-04-01

**Authors:** Juri Juksar, Rachel Mijdam, Sabine Bosman, Alexander van Oudenaarden, Françoise Carlotti, Eelco J. P. de Koning

**Affiliations:** ^1^ Hubrecht Institute-KNAW (Royal Netherlands Academy of Arts and Sciences), Utrecht, Netherlands; ^2^ Department of Internal Medicine, Leiden University Medical Center, Leiden, Netherlands

**Keywords:** pancreas, organoid, NEUROG3, delamination, differentiation

## Abstract

Diabetes mellitus is characterized by the loss of pancreatic insulin-secreting β-cells in the Islets of Langerhans. Understanding the regenerative potential of human islet cells is relevant in the context of putative restoration of islet function after damage and novel islet cell replacement therapies. Adult human pancreatic tissue can be cultured as three-dimensional organoids with the capacity for long-term expansion and the promise of endocrine cell formation. Here, we characterize the endocrine differentiation potential of human adult pancreatic organoids. Because exocrine-to-endocrine differentiation is dependent on the expression of Neurogenin 3 (NEUROG3), we first generated NEUROG3-inducible organoid lines. We show that doxycycline-induced NEUROG3 expression in the organoids leads to the formation of chromogranin A positive (CHGA+) endocrine progenitor cells. The efficiency of this differentiation was improved with the addition of thyroid hormone T3 and the AXL inhibitor R428. Further, compound screening demonstrated that modifying the pivotal embryonic endocrine pancreas signalling pathways driven by Notch, YAP, and EGFR led to increased NEUROG3 expression in organoids. In a similar fashion to embryonic development, adult ductal cells delaminated from the organoids after NEUROG3 induction. Thus, mechanisms in islet (re)generation including the initiation of endocrine differentiation and delamination can be achieved by NEUROG3 induction.

## Graphical Abstract

**Figure:**
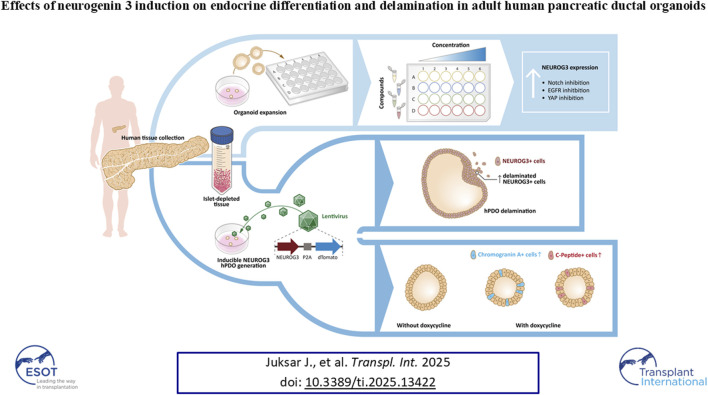

## Introduction

Diabetes mellitus results from the dysfunction and/or loss of insulin-producing β-cells [1]. The low cell turnover and regenerative capacity of the endocrine cells hamper the restoration of a sufficient functional islet mass [2]. Safe and reliable strategies for β-cell replacement are investigated as potential therapeutic options, including 1) the transplantation of donor islets or stem cell-derived cellular products [3–5] and 2) the induction of endogenous regeneration either by beta cell replication or (trans)differentiation [6–9].

Harnessing the endogenous regenerative capacity of the pancreas could yield treatment options applicable to different forms of diabetes. The regenerative capacity of the human pancreas was observed through beta cell mass regulation happening at different stages of life, including early postnatal life, pregnancy, and obesity [10, 11]. The source of new beta cells however remains elusive, though it has been proposed that new endocrine cells could form from pancreatic ducts, due to the close relationship of adjacent ducts and islets [11, 12] and their shared developmental origin [13]. Recently, an LGR5^+^ tripotent progenitor cell, capable of differentiating into all three pancreatic cell lineages, was identified in the human fetal pancreas [14].

Although the generation of endocrine cells from adult ductal tissue is controversial, with some mouse lineage tracing studies showing β-cell neogenesis, while others showing no external contribution to the islet cell composition [15–21], adult pancreatic duct organoids (PDOs) have shown that they can be differentiated *in vitro* and *in vivo* towards an endocrine-like state [22–25]. While the rodent studies have demonstrated that under certain conditions, such as pancreatic injury or metabolic stress, β-cells can regenerate from progenitor cells within the pancreatic duct, human studies have not consistently replicated these findings. The adult human pancreas exhibits limited β-cell regeneration from ductal progenitors, possibly due to species-specific differences in cellular plasticity and regenerative mechanisms [26].

In addition, PDOs possess the capacity for long-term expansion *in vitro* without losing the characteristics of their tissue of origin, making them an attractive source for duct cells [27]. However, under current conditions they struggle to generate functional glucose-responsive endocrine cells, nor do they exhibit delamination to form *de novo* islet-like clusters.

In embryogenesis, the key regulator of ductal cell differentiation towards an endocrine cell phenotype is Neurogenin 3 (NEUROG3), which leads to the downstream activation of a number of crucial genes that drive pancreatic endocrine cell formation [28]. Its absence during development leads to an almost total loss of islet cells. By harnessing this knowledge, genetic modification of human exocrine cell cultures through combining NEUROG3 with hallmark beta cell transcription factors PDX1 and MAFA, converts the cells towards an endocrine islet-like fate [29], showing that pancreatic duct cells have the capacity for endocrine differentiation. The question remains, however, whether ductal cells possess an intrinsic capacity for the initiation of differentiation without transgenic manipulation and whether they follow a predictable differentiation timeline.

To that end, we report that inhibition of EGFR, Notch, and YAP are crucial signalling pathways involved in regulating NEUROG3 expression in human PDOs (hPDOs). Upon differentiation, hPDO cells undergo delamination from the epithelium and enter the surrounding matrix. hPDOs form CHGA+ endocrine progenitor-like cells upon NEUROG3 expression, that are responsive to manipulation by thyroid hormone and AXL inhibition. Together, this study reveals a capacity for early differentiation initiation in hPDOs further building on our understanding of human pancreatic duct cell biology.

## Materials and Methods

### Generation and Expansion of hPDOs

Human pancreatic islet-depleted tissue for hPDO generation was obtained after islet isolation from donor pancreas after density gradient separation, procured via the Eurotransplant multiorgan donation program at the Leiden University Medical Center (Supplementary Table S1). It was only used in experiments if research consent was present, according to Dutch national laws. The islet-depleted tissue was CMRL-1066 medium (Cellgro) supplemented with 10% human serum (Blood bank LUMC, Leiden) and 1% penicillin/streptomycin (Invitrogen) was used to culture the islet-depleted tissue before hPDO generation.

Before hPDO generation, the islet-depleted tissue was first washed three times with Dulbeccos phosphate buffered saline (DPBS; Thermo Fisher Scientific), and passed twice through a syringe with a 21 g needle (BD Microlance 3). The dissociated tissue was then resuspended in Matrigel (Growth Factor Reduced Basement Membrane Matrix; Corning) and plated in 6-well plates (CELLSTAR^®^), upside down at 37°C for 15 min, with 5% CO2. Once Matrigel solidified, 2 mL of culture media was added per well.

hPDO expansion was adapted from our previous publications [24, 25]. Culture media consisted of advanced Dulbecco’s Modified Eagle Medium/Ham’s F-12 (advanced DMEM/F-12; Thermo Fisher Scientific) supplemented with 10 mM HEPES (Thermo Fisher Scientific), 1% GlutaMAX (Thermo Fisher Scientific), 1% penicillin/streptomycin (Thermo Fisher Scientific), 2% B27 without vitamin A (Invitrogen), 10% R-Spondin1 conditioned media (prepared in-house), 1% Noggin conditioned media (U-Protein Expressed BV), 10 mM nicotinamide adenine dinucleotide (NAD, Sigma Aldrich), 10 mM PGE2 (Tocris), 1 mM N-acetylcysteine amide (NAC; Sigma Aldrich), 3 µM CHIR99021 (Axon Medchem), 10 nM Gastrin (Tocris), 5 µM A83-01 (Axon Medchem), 50 ng/mL human epidermal growth factor (EGF; PeptroTech), 100 ng/mL fibroblast growth factor 10 (FGF10; PeproTech) and 100 μg/mL primocin (InvivoGen). The culture medium was refreshed two to three times a week depending on number of hPDO’s.

Once a week, hPDOs were passaged at a 1:2 ratio by mechanically disrupting them using a glass pipette. The cells were then centrifuged at 300 g for 4 min at 4°C, resuspended in Matrigel, and plated as described previously. Following passage, the culture media was supplemented with 10 µM ROCK inhibitor (Y-27632; Abmole) for 24 h.

For suspension culture, hPDOs were passaged as previously mentioned and incubated in Cell Recovery solution for 30 min on ice. The cells were then plated in low retention plates in regular culture media supplemented with ROCK inhibitor.

### Generation of NEUROG3-hPDO Lines by Lentiviral Transduction

A previously published doxycycline-inducible NEUROG3-P2A-dTomato plasmid was used to generate the transgenic hPDOs [47]. Three hPDO lines derived from different donors were lentivirally transduced following the protocol described by Broutier et al [48]. Lentiviral transduction was performed on small clumps of cells, after dissociation with TrypLE (TrypLE Express; Life Technologies). Lentiviral transduction was performed on hPDO’s at passage 5

### Endocrine Differentiation of hPDOs

Small molecule-based differentiation was performed on hPDO’s after 5 passages in expansion culture. For differentiation experiments, hPDOs were plated in 6 or 12-well plates and expanded for 6 days prior to inducing endocrine differentiation. Before adding the differentiation medium, the wells were washed twice by incubating them with PBS for 5 min at 37°C. For differentiation experiments using NEUROG3-hPDOs (passages 8–10), NEUROG3 expression was initiated by 24 h incubation with doxycycline (1 μg/mL) in basal differentiation medium. To facilitate spontaneous differentiation, a cytokine-free basal differentiation medium was used, containing advanced DMEM/F12 (Invitrogen), 10 mM HEPES (Life Technologies), 1% GlutaMAX (Life Technologies), 1% penicillin/streptomycin (Life Technologies), 1 mM NAC (Sigma Aldrich) and 2% B27 plus vitamin A (Invitrogen). The concentrations and compounds that were used in the differentiation protocols are described in Supplementary Table S2 and were chosen based on previously published (i)PSC beta cell differentiation protocols [3, 4]. hPDOs were subsequently harvested at the indicated time point.

### RNA Isolation and qRT-PCR

RNA was isolated using the GenElute™ Mammalian Total RNA Miniprep Kit (Sigma Aldrich), followed by cDNA synthesis using the iScript™ cDNA Synthesis Kit (Bio-Rad) with 500 ng of RNA.

Quantitative real-time polymerase chain reaction (qRT-PCR) was conducted following the manufacturer’s instructions. Briefly, 1 µL of primers (Supplementary Table S3), 5 µL of SYBR green (Bio-Rad), and 5 µL of cDNA were added to a 384-well plate in triplicate. Gene expression was normalized to RPLP0 and GADPH as housekeeping genes, and the relative expression was calculated using the 2^(-ΔCq) method.

### Flow Cytometry

hPDOs were harvested in cold DPBS and centrifuged at 300 g for 4 min at 4°C. Subsequently, they were incubated in 2 mL of TrypLE Express (Thermo Fisher Scientific) for 15 min at 37°C. The single cells obtained were then washed with cold DPBS before being used in downstream analysis.

Dissociated cells were stained with the LIVE/DEAD™ 405 Violet Fixable Dead Cells Stain Kit (Invitrogen). For intracellular staining, cells were fixed and permeabilized using the eBioscience™ Foxp3/Transcription Factor Staining Buffer Set (Invitrogen) following the manufacturer’s instructions. Subsequently, cells were stained with primary, secondary, or conjugated antibodies (Supplementary Table S4) for 30 min at room temperature or overnight at 4°C. Flow cytometry analysis was performed using the BD FACSCanto™ II and the BD LSRFortessa™ X20 flow cytometers (BD Biosciences), and data were analyzed using FlowJo v7.6.5 software.

### Microscopy and Immunohistochemical Staining

For immunohistochemical analysis, organoids were fixed in 4% paraformaldehyde (PFA) and embedded in paraffin. Tissue sections (4 μm) were cut and incubated with primary antibodies at 4°C overnight and with secondary antibodies at room temperature for at least 2 h (Supplementary Table S4).

Fluorescent images were captured with a Leica SP8 confocal microscope. Images were further processed with LAS AF software (Leica). Brightfield pictures were captured with a Leica DMIL microscope and processed with Leica LAS AF lite software or using the EVOS FL Digital Inverted Microscope (Invitrogen).

### Live Imaging of Delamination

NEUROG3-hPDOs were plated in 50 µL of Matrigel per condition in Cellview cell culture dishes (Greiner). Doxycycline (1 μg/mL) was added 6 hours before imaging, and dTomato expression was monitored over 48 h. NucRed™ Dead 647 ReadyProbes™ Reagent (ThermoFisher Scientific) was used as a viability stain, following the manufacturer’s protocol. Imaging and analysis were conducted using the Leica SP8 confocal microscope and LAS X software.

### Statistical Analysis

Values are shown as mean ± standard deviation (SD). The Mann-Whitney U test was used as a non-parametric test. P values < 0.05 were considered significant. GraphPad Prism was used for statistical analysis. qPCR data calculated and presented as ΔCt.

## Results

### Compound Screening Reveals EGF, Notch, and YAP as Pathways Affecting NEUROG3 Expression in hPDOs

Primary human islet depleted tissue from 7 donors consisted of only 0%–12% non-exocrine (non-ductal and non-acinar) cells (Supplementary Figure S1A; Supplementary Table S1). After 2 passages the majority of hPDO cells were positive for the ductal marker cytokeratin 19 and the epithelial marker E-cadherin and negative for the endocrine markers insulin and glucagon (Supplementary Figures S1B, C).

**FIGURE 1:**
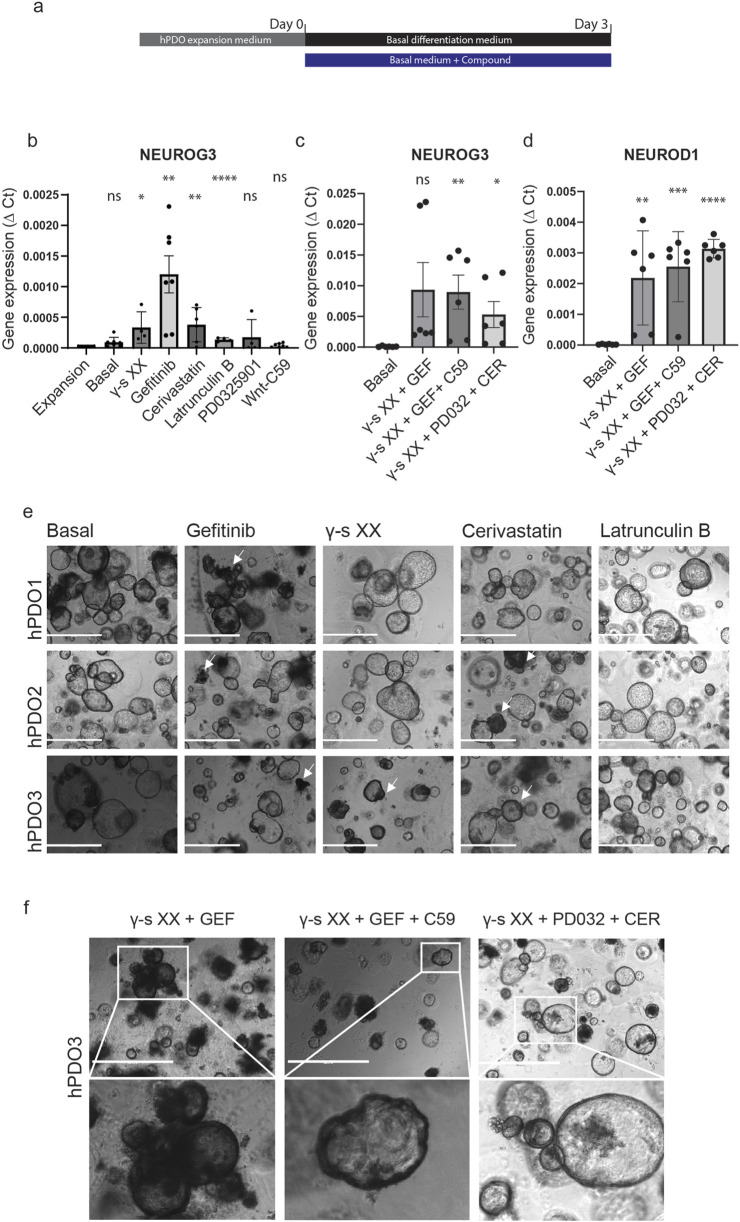
Compound screening reveals EGF, Notch, and YAP as pathways affect NEUROG3 expression in hPDOs. **(A)** Schematic overview of experimental setup – samples were collected on day 0 after hPDO expansion and 3 days after differentiation. The black bar represents basal differentiation medium only and the blue bar represents basal differentiation medium with compounds added. Basal differentiation medium consisted of Advanced DMEM/F12 with 10 mM HEPES, 1% GlutaMAX, 1% penicillin/streptomycin, 1 mM NAC and 2% B27 plus vitamin A (Basal). The list of compounds and concentrations used can be found in Supplementary Table S2. hPDO’s at passage 5 were used for differentiation. **(B)** qRT-PCR analysis of NEUROG3 expression in hPDOs treated with single compounds. Data are presented as mean with standard deviation and significance calculated against hPDO in expansion medium (Expansion). **(C)** qRT-PCR analysis of NEUROG3 expression in hPDOs upon combinatorial treatment. Data are presented as mean with standard deviation and significance calculated against basal differentiation medium (Basal). GEF- gefitinib, CER – cerivastatin, PD032 – PD0325901, C59 – Wnt-C59, γ-s XX – gamma secretase inhibitor XX. **(D)** qRT-PCR analysis of NEUROG3 downstream target NEUROD1 expression in hPDOs upon combinatorial treatment. Data are presented as mean with standard deviation and significance calculated against basal differentiation medium (Basal). **(E)** Brightfield microscopy images of 3 hPDO lines (hPDO1, hPDO2, hPDO3) upon single compound treatment. White arrowheads denote denser morphological areas upon treatment. Scalebar 1,000 µm. **(F)** Brightfield microscopy images of hPDO3 line with combinatorial compound treatment. Inset images shown at higher resolution below. Scalebar 1,000 μm. n = 2 repeats on 3 hPDO lines.

To evaluate the signalling pathways involved in NEUROG3 activation, we performed a compound screening using islet-depleted tissue-derived hPDO lines. To ensure that only consistent effects across diverse lines were captured, six hPDO lines were pooled after compound treatment. Gene expression analysis revealed an upregulation in NEUROG3 expression upon inhibition of Notch, EGF and YAP signalling (Supplementary Figure S2). Based on these results, we selected the three most responsive hPDO lines for subsequent experiments (Figure 1A). hPDOs were cultured in a basal medium, with added supplements for 3 days before harvesting the organoids for gene expression analysis. Inhibition of Notch signalling by gamma-secretase inhibitor XX (γ-S XX), led to a low but consistent upregulation of NEUROG3 expression in hPDOs (Figure 1B). A similarly low but consistent effect was seen by the addition of Latrunculin B, an inhibitor of actin polymerization. Supplementing hPDOs with the EGF receptor (EGFR) inhibitor Gefitinib showed the highest upregulation of NEUROG3 with the highest variability between different hDPO lines. Finally, the addition of the mevalonate and YAP pathway inhibitor cerivastatin increased the expression of NEUROG3 in hPDOs. Although all singular compounds did not show a strong upregulation in NEUROG3, they showed a consistent and significant effect on its expression.

The effect could be further strengthened by combining the compounds into a single 3-day treatment (Figure 1C). Combining Notch and EGFR inhibition (γ-S XX + Gefitinib) resulted in a significant upregulation of NEUROG3 expression when compared to the compounds separately suggesting a synergistic effect to inhibition of both pathways. The further addition of the Wnt pathway inhibitor C59 resulted in a more consistent pattern of NEUROG3 upregulation between different hPDO lines. To verify NEUROG3 expression we also analysed the expression of its downstream target NEUROD1. While there was no expression detected in single compound screens (data not shown), we detected significant upregulation of NEUROD1 in combined compound screens (Figure 1D). By combining Notch, MAPK, and mevalonate pathway inhibition we detected the most consistent upregulation in NEUROD1, though its expression of NEUROG3 was not as high as in other conditions. In addition, while the morphology of hPDOs remained consistent during treatment, some hPDOs had a dark phenotype and formed cellular protrusions and a thickened organoid lining (Figures 1E, F, white arrowheads). However, with the current experimental conditions, we did not detect protein expression of CHGA (data not shown). Combined, our data shows that Notch, EGF, and YAP play an important role in hPDO differentiation in activating NEUROG3 and its downstream target NEUROD1 in adult tissue-derived cells.

### Doxycycline-Induced Expression of NEUROG3 in hPDOs Leads to the Expression of Downstream Targets NEUROD1, NKX2.2, and PAX4

**FIGURE 2:**
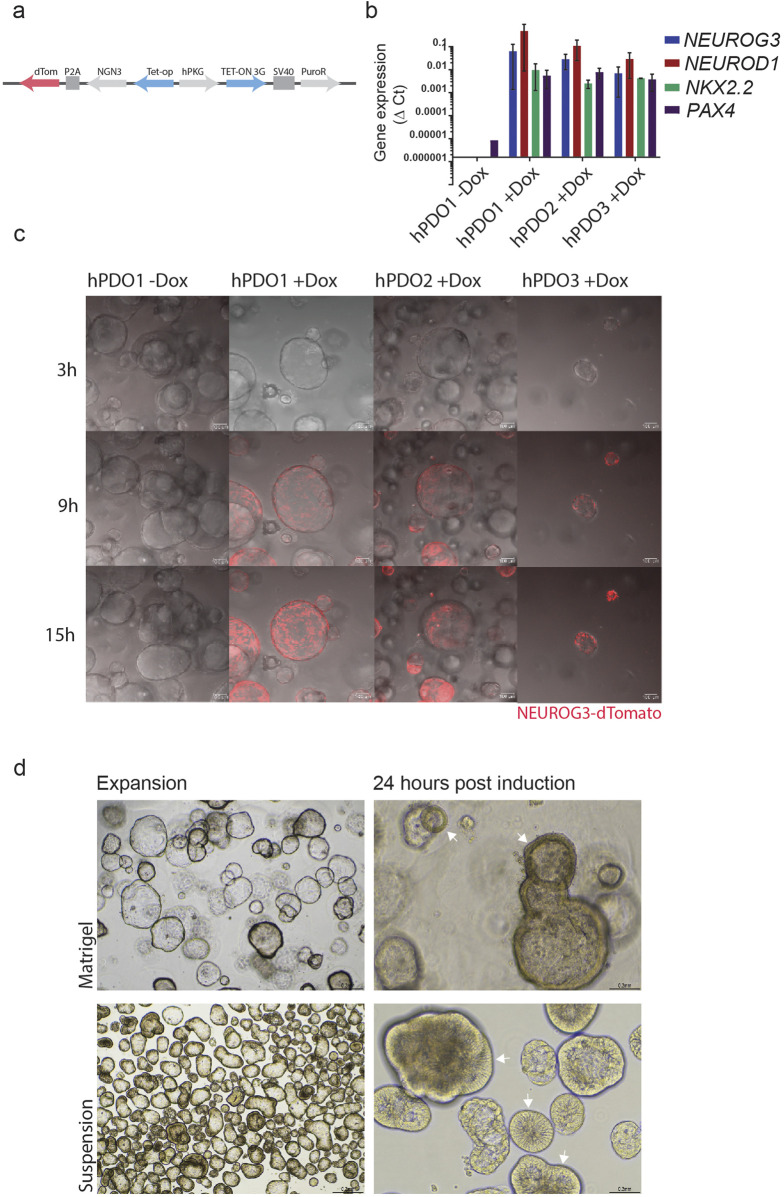
Doxycycline-induced expression of NEUROG3 in hPDOs leads to the expression of downstream targets NEUROD1, NKX2.2, and PAX4 **(A)** Overview of NEUROG3-P2A-dTomato construct used in transduction of hPDO lines at passage 5. **(B)** qRT-PCR analysis of NEUROG3 and its downstream targets NEUROD1, NKX2.2 and PAX4 upon doxycycline induction. Data are presented as mean with standard deviation. **(C)** Time-course analysis of NEUROG3 expression in NEUROG3-hPDOs by live confocal imaging. Snapshots taken at 3, 9, and 15 h post-induction. **(D)** Brightfield microscopy images of hPDOs showing thickening of cell walls upon NEUROG3 induction in expansion and 24 h post-induction in Matrigel and suspension culture (arrowheads). Top row hPDOs embedded in matrigel, bottom row cultured in suspension. n = 2 repeats on 3 hPDO lines.

To further assess the endocrine differentiation potential of hPDOs, we used lentiviral transduction to generate 3 transgenic hPDO lines with a doxycycline-inducible NEUROG3 construct – NGN3-hPDO (Figure 2A). NEUROG3 expression upon doxycycline induction was validated by qPCR which showed a robust expression of NEUROG3 (Figure 2B). Expression of the NEUROG3 downstream target genes NEUROD1, NKX2.2, and PAX4 was robustly upregulated in all three lines 3 days after induction, validating the presence of a functional NEUROG3 protein.

Finally, by tracking the expression of dTomato, the time course analysis revealed NEUROG3 expression starts approximately 9 h post-induction, reaching its peak by 15 h post-induction (Figure 2C, Supplementary Video S1). Additionally, we observed a rapid shift in the structure of hPDOs by 24 h post induction, when they adopt a denser morphology with a thicker epithelial layer (Figure 2D). These changes were especially noticeable during suspension culture and occurred rapidly at the onset of NEUROG3 expression starting within the first 15–24 h post-induction (Supplementary Video S2).

### NEUROG3 Expression in hPDOs Initiates a Step-Wise Expression of Endocrine Differentiation Genes

**FIGURE 3:**
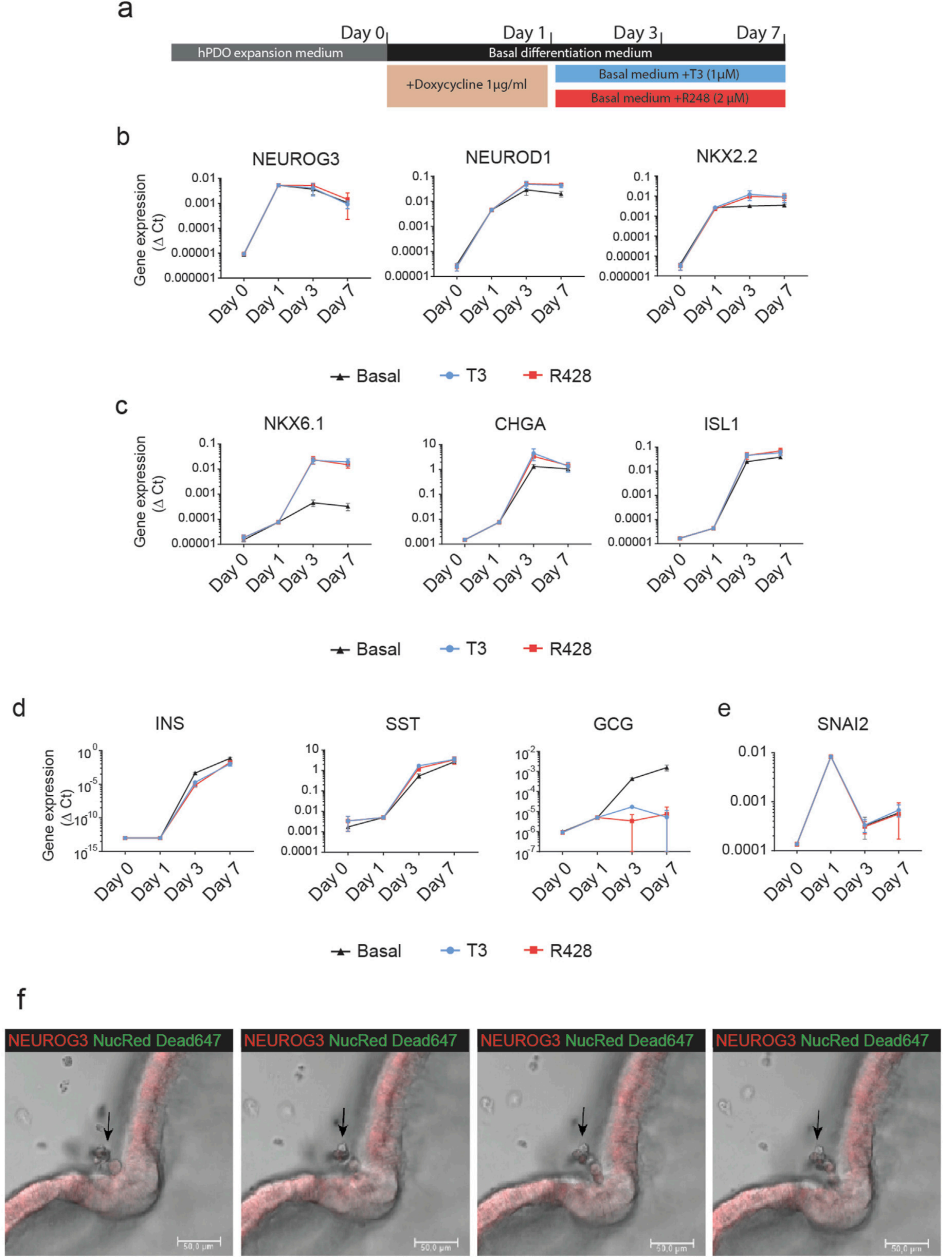
NEUROG3 expression in hPDO’ initiates a step-wise expression of endocrine differentiation genes **(A)** Schematic overview of NEUROG3-hPDO differentiation. Organoids were passaged and expanded for 6 days before a 24-h transient doxycycline treatment. This was followed by a 7-day differentiation period in basal differentiation media only, or with addition of T3 or R428. Cells were collected for qRT-PCR analysis at end of expansion, and 24 h, 3 days and 7 days. **(B)** qRT-PCR analysis of early endocrine genes in basal differentiation medium or upon treatment with T3 or R428 over the differentiation period. **(C)** qRT-PCR analysis of endocrine progenitor genes peaking at day 3 of differentiation. **(D)** qRT-PCR analysis of pancreatic hormone expression in hPDOs upon NEUROG3 induction. **(E)** qRT-PCR analysis of the EMT marker SNAI2 which was transiently expressed 24 h after induction. **(F)** Time-course confocal images of invagination of hPDO epithelium and outwards delamination of single live cells from the NEUROG3-hPDOs at 24, 25, 26, and 26.5 h post NEUROG3 induction. Red - NEUROG3-P2A-dTomatol, Green – Nuclear Red Dead 647 viability dye. n = 2 repeats on 3 hPDO lines. Data are presented as mean with standard deviation. qPCR data are presented as Δ-Ct.

To characterize the effect of ectopic NGN3 expression on the endocrine differentiation of hPDOs, we performed qRT-PCR analysis to measure the expression levels of genes involved in embryonic endocrine pancreas development over a 7-day differentiation period. Gene expression was measured at expansion (Day 0) and at 24 h, Day 3 and Day 7 of the differentiation protocol (Figure 3A). Within the first 24 h, we observed a significant upregulation of NEUROD1 and NKX2.2 concomitantly with NEUROG3 (Figure 3B). These early endocrine genes are direct downstream targets of NEUROG3 and they reached their peak by day 3 of differentiation and remained stably expressed until day 7. By day 3, we observed a marked increase in NKX6.1, CHGA, and ISL1, which we call late endocrine genes in this manuscript (Figure 3C). These genes showed a very low increase in expression 24 h after induction, but a major upregulation by day 3. A similar pattern can be observed in the expression of insulin (INS) and somatostatin (SST) (Figure 3D). Interestingly, glucagon (GCG) expression was not significantly impacted by the NEUROG3-induced differentiation.

To assess the impact of pro-endocrine differentiation compounds on NEUROG3-induced differentiation, we supplemented the basal media with thyroid hormone T3 and AXL inhibitor R428. These treatments enhanced the expression of the β-cell-specific gene NKX6.1 compared to basal media alone, indicating a supportive role in driving β-cell-like differentiation, as previously described in pluripotent stem cells [3]. Additionally, we observed a reduction in GCG expression, suggesting a potential shift away from α-cell-like fate. These findings support the role of T3 and R428 in promoting a β-cell-like gene expression profile in hPDOs.

An important aspect of embryonic endocrine pancreas development is the delamination of NEUROG3+ endocrine progenitor cells from the ductal lining. Interestingly, upon NEUROG3 induction, the EMT marker SNAI2 was transiently upregulated at the 24-h mark during differentiation, before being downregulated by day 3 (Figure 3E). Live time-course imaging confirmed delamination induction which coincided with the expression of SNAI2 (Figure 3F, arrowhead; Supplementary Video S3). NEUROG3-hPDOs formed invaginated delamination sites with thickened cell walls, from which single live cells exited the lining of the organoid and entered the surrounding matrigel. This continued for up to 48 h with gaps that formed within the NEUROG3-hPDO lining (Supplementary Video S4).

Combined, our data show that doxycycline-induced NEUROG3 expression initiated endocrine differentiation in hPDOs marked by expression of endocrine marker genes (NKX6.1, CHGA, ISL1) and induced delamination concomitantly with expression of EMT marker SNAI2.

### NEUROG3-hPDOs Form CHGA+ Endocrine Progenitor-Like Cells That Respond to T3 and R428 Treatment

**FIGURE 4:**
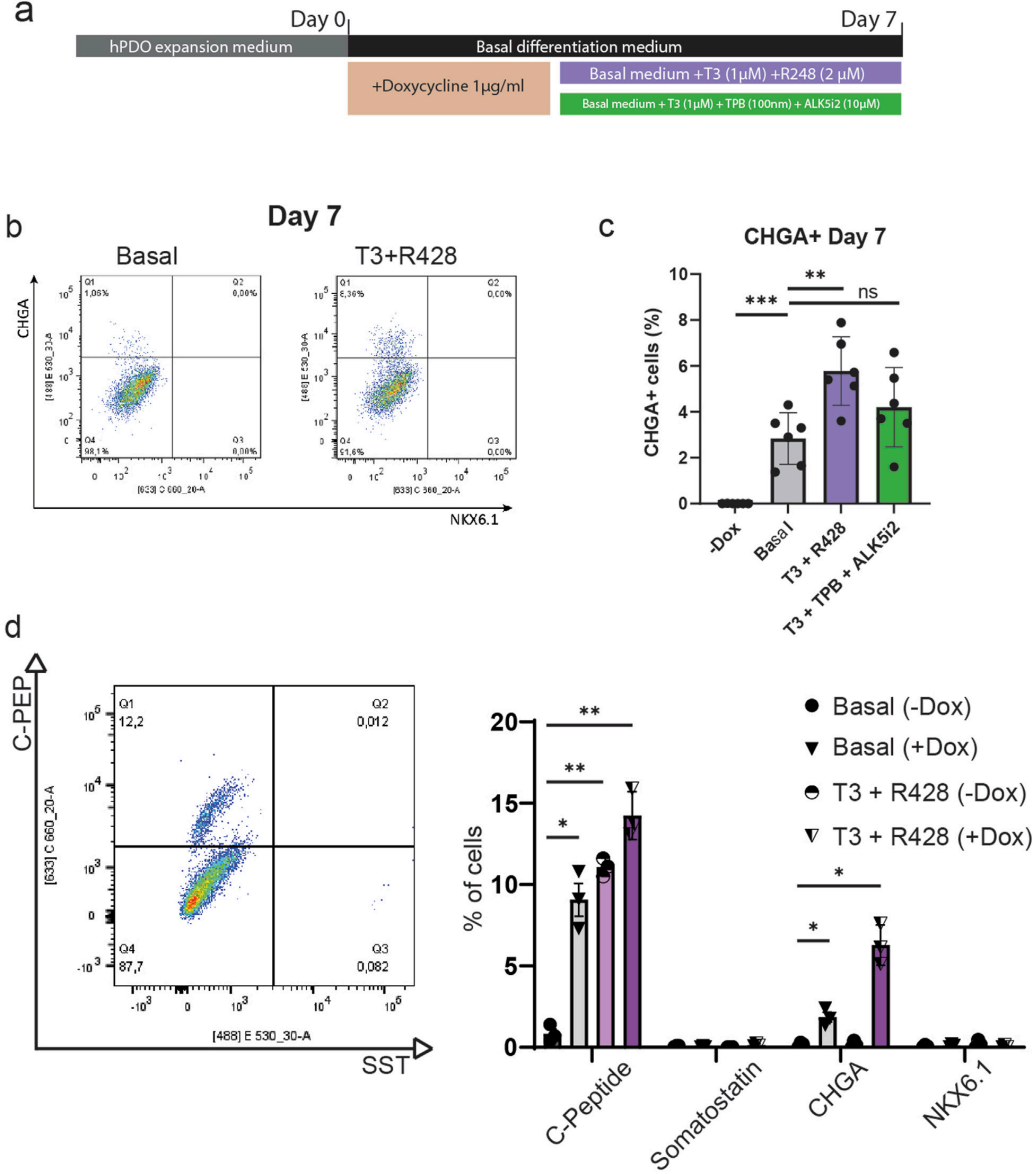
NEUROG3-hPDOs form CHGA+ endocrine progenitor-like cells that respond to T3 and R428 treatment. **(A)** Schematic overview of NEUROG3-hPDO differentiation upon treatment with compounds promoting beta cell fate – T3 + R428 and T3 + TPB + ALK5i2. Samples were collected on day 7. **(B)** Flow cytometry analysis of beta cell progenitor markers CHGA and NKX6.1 in basal differentiation media with addition T3 and R428. **(C)** Comparative analysis of CHGA+ cell differentiation efficiency upon treatment after NEUROG3 induction. n = 2 repeats on 3 hPDO lines. **(D)** Analysis of NKX6.1, CHGA, C-peptide (C-PEP) and somatostatin (SST) expression at protein level, n = 3. Data are presented as mean with standard deviation.

To validate whether doxycycline-induced NEUROG3 expression can lead to the formation of an endocrine progenitor-like population we performed flow cytometry analysis for the pan-endocrine marker gene CHGA. We found that upon doxycycline induction, roughly 2%–4% of NEUROG3-hPDO cells are CHGA+ (Figures 4A, B), alluding to the existence of a subpopulation of hPDO cells that can progress into an early endocrine progenitor-like state. While we noted the presence of CHGA+ cells, they did not express the *bona fide* beta cell marker NKX6.1 at the protein level.

To investigate whether this differentiation process can be improved, we applied two commonly used combinations of compounds used in pluripotent stem cell-derived beta cell differentiation protocols – the thyroid hormone T3 and AXL inhibitor R428, or T3, protein kinase C (PKC) activator TPB and ALK5 (TGF-β receptor) inhibitor II. By applying these factors for 7 days, we show that T3 and R428 can significantly increase (up to 4%–8%) the number of CHGA+ cells but had no effect on protein expression of NKX6.1 (Figures 4B, C). Finally, by targeting rho-associated protein kinase (ROCK) with the inhibitor Y-27632, we observed a significant decrease in CHGA+ cells and retention of pre-induction morphology (Supplementary Figure S3). Despite the low overall differentiation efficiency observed at the population level, the consistent emergence of endocrine-like cells suggests the presence of a responsive subpopulation, highlighting an important avenue for future studies in the context of pancreas regeneration that are aimed at optimizing differentiation protocols and investigating cellular plasticity within hPDO cultures.

To verify hormone expression at the protein level, hPDO’s treated with T3 and R428 were analysed by flow cytometry for C-Peptide (C-PEP) and somatostatin (SST) on day 7 (Figure 4D). We observed a marked increase in C-Peptide expression upon NEUROG3 induction (ca 9%), which was further improved by addition of T3 and R428 (ca 14%). Importantly, treatment with T3 and R428 without NEUROG3 induction increased the number of C-PEP+ cells to 11%, showing that a sub-population of hPDO cells can be induced to express insulin without need for transgenic NEUROG3 expression. The C-PEP+ cells however lacked co-expression of NKX6.1 which is generally considered to be required for *de novo* beta cells. Additionally, while SST gene expression was increased, we found no significant expression of somatostatin at a protein level using flow cytometry.

## Discussion

As β-cells arise from a multipotent progenitor in the ductal compartment during embryonic development, the endocrine differentiation of adult pancreatic duct cells has long been considered a potential source for *de novo* β-cells [20, 30]. While duct cells have been shown to have the capacity to form endocrine-like cells *in vitro* (as cell lines, explants, or organoids), their (trans)differentiation efficiency, post-differentiation functionality, or *bona fide* endocrine identity often remain elusive.

Using compound screening, we were able to determine three key signalling pathways which directly affect NEUROG3 expression in cultured hPDOs–Notch, YAP, and EGFR. The Notch signalling pathway and its downstream target HES1 have been shown to employ a multi-layered control over endocrine differentiation, in part by HES1 binding to the promoter of NEUROG3 [31]. Similarly, YAP has been shown to be downregulated following endocrine fate specification from multipotent pancreatic progenitor cells *in vivo* and *in vitro* [32, 33]. This is likely due to YAP being a regulator of transcription factors that establish the multipotency and expansion of pancreatic progenitors, thus placing them in a crucial role in establishing whether a progenitor cell undergoes proliferation or differentiation [34]. Harnessing this, the downregulation of YAP via small molecules *in vitro* during pluripotent stem cell-derived endocrine differentiation has been shown to enhance the generation of endocrine progenitors and stem cell-derived beta cells [32, 33].

EGFR signalling has been linked to cellular fate control during pancreatic organogenesis by regulating the apicobasal polarity of NEUROG3+ endocrine progenitors in a ligand-specific manner [35]. One of these ligands, betacellulin, has been linked to the activation of PI(3)K and Rac1, which in turn leads to the inhibition of aPKC and Notch, further upregulating NEUROG3. These results warrant further investigation into the context and ligand-dependent effects of EGFR inhibition or activation. In addition, EGFR inhibition leads to the upregulation of NEUROG3 and enteroendocrine cell formation in intestinal organoids when used in combination with Notch and Wnt inhibition, by inducing cell cycle exit in Lgr5+ adult intestinal stem cells [36]. Importantly, the combinatorial effect of targeting multiple pathways is key in inducing a high enough expression of NEUROG3 in hPDOs for it to induce its downstream target NEUROD1. By combining inhibition of Notch, YAP, and EGFR, we likely ameliorate the block on the transcriptional control of NEUROG3 and push the cells towards an endocrine differentiation state, leading to the activation of NEUROG3 and NEUROD1. This consistency underscores the potential role of these pathways in regulating key transcriptional networks associated with endocrine differentiation. Further investigation is warranted to fully elucidate their contributions to lineage commitment in hPDO’s and the viability of small molecule inhibitors to drive this differentiation by themselves.

Given that pancreatic ductal cells are the cells of origin for pancreatic ductal adenocarcinoma (PDAC), manipulating key signalling pathways such as EGFR, Notch, and YAP carries potential oncogenic risks [37]. EGFR and YAP are well-established drivers of PDAC when overactivated, and their inhibition has been explored as a therapeutic strategy [38–40]. Notch signalling, however, has a more complex, context-dependent role, acting as both a tumour promoter and suppressor depending on the specific receptor and tumour stage [41]. While we leveraged Notch inhibition to promote endocrine-like differentiation in hPDOs, we acknowledge its dual role in PDAC progression. Future studies should explore long-term effects, assess potential transformation risks, and refine pathway modulation to ensure safe and effective use of hPDOs as research tools.

By using a doxycycline-inducible NEUROG3-hPDO system, we were able to show that early differentiation can be triggered within human adult ductal cells, leading to the formation of a CHGA+ subpopulation of endocrine progenitor-like cells. Interestingly, activation of NEUROG3 was enough to induce not only its direct downstream target genes but also included upregulation of the wider network of genes involved in endocrine fate determination, including NKX6.1, ISL1, and CHGA within the cultured duct cells. Similarly, the expression of hormone genes insulin and somatostatin was positively upregulated after 7 days of differentiation. However, further validation of protein expression data for endocrine-specific genes is essential to accurately assess the differentiation potential of hPDOs.

The presence of CHGA+ cells could further be enhanced by the addition of T3 and R428 to the differentiation media following NEUROG3 induction. The thyroid hormone T3 has been linked to pancreatic development and differentiation by promoting beta cell differentiation and maturation in pluripotent stem cell-derived islet clusters [3, 4, 42, 43]. Similarly, hypothyroidism has been linked to impaired C-peptide secretion in mice post-transplantation of pancreatic progenitors [44]. Targeting the tyrosine kinase receptor AXL by R428 has been linked with the induction of the beta cell maturation marker MAFA in pluripotent stem cells [3]. As T3 and R428 have been previously linked to pancreatic development, their application in adult-like human pancreatic ductal organoids (hPDOs) offers new insights into endocrine differentiation within a postnatal tissue context.

Delamination is a crucial step in the differentiation process during embryonic pancreas development, in which multipotent cells within the lining of the developing ductal system undergo a partial epithelial-to-mesenchymal transition and exit the ductal lining [45]. Apico-basal polarity is a crucial regulator of endocrine differentiation via a regulatory circuit of ROCK-nmMyoII, NEUROG3, and Notch signalling [46]. By disrupting this feedback mechanism by inhibiting ROCK, we show a distinct decrease in CHGA+ cells after induction and a lack of morphological condensation, leading us to believe that delamination in hPDOs is governed by the pre-established principles found *in vivo*.

Importantly, while the CHGA+ cells exhibit early endocrine differentiation traits, protein analysis of insulin and somatostatin, which were highly expressed on a transcriptomic level, showed that around 14% of hPDO cells expressed C-peptide – a proxy for insulin. These cells, however, lacked the co-expression of NKX6.1 protein which is crucial for the fate specification of *bona fide de novo* beta cells.

A critical step in understanding the differentiation process in hPDOs involves characterizing the delaminating cell population following endocrine induction. While our findings demonstrate the initiation of endocrine-like differentiation, the precise identity, maturation status, and fate of these delaminated cells remain unclear. Isolating and profiling these cells pose significant technical challenges due to their dispersed nature and the lack of established surface markers specific to this transitioning population. Future studies employing advanced cell-sorting technologies, live-cell imaging, and single-cell transcriptomic analyses will be essential for gaining deeper insights into their molecular signatures and differentiation potential. Addressing these challenges will be pivotal for elucidating the full spectrum of cellular plasticity within adult pancreatic ductal organoids.

Taken together, our data suggests that hPDO cells can be induced to initiate the early processes crucial for endocrine differentiation and they are susceptible to further stimulation to drive them towards an endocrine fate post NEUROG3+ induction. The emergence of CHGA+ cells points towards the presence of a subpopulation of cells within hPDOs with a higher capacity for endocrine differentiation, and this is likely not an inherent characteristic of all hPDOs, and by extension, pancreatic duct cells. As such, further study into the heterogeneity of hPDOs and their tissue of origin could yield crucial information into the origin of the CHGA+ subpopulation.

## Data Availability

The raw data supporting the conclusions of this article will be made available by the authors, without undue reservation.

## Supplementary Material

SUPPLEMENTARY TABLE S1 Organ donor characteristics.

SUPPLEMENTARY TABLE S2 Compounds used in differentiation experiments.

SUPPLEMENTARY TABLE S3 Primers used in qRT-PCR analysis.

SUPPLEMENTARY TABLE S4 List of antibodies.

SUPPLEMENTARY FIGURE S1 Characterisation of islet-depleted tissue and hPDO's after 2 expansion passages **(A)** Flow cytometry analysis of islet-depleted tissue (IDT) for exocrine markers GP2 (acinar) and CD59 (exocrine) before culturing. The percentage of acinar cells (GP2+/CD59+) or GP2- exocrine cells (GP2-/CD59+) is represented in the table, showing that the majority of islet-depleted tissue is exocrine (GP2+/CD59+ and GP2-/CD59+). **(B)** hPDO's after 2 passages resemble cystic structures with widespread expression of ductal marker CK19 and epithelial marker E-Cadherin. **(C)**_hPDO's do not express the acinar protein amylase (AMY) or the endocrine hormones glucagon (GCG) or insulin (INS) after 2 passages in expansion medium.

SUPPLEMENTARY FIGURE S2 Compound screen of possible NEUROG3 effectors in human adult pancreatic ductal organoids (A) qRT-PCR analysis of 6 pooled hPDO lines per condition after treatment with compounds and small molecules affecting Notch, EGFR, YAP, mevalonate, Wnt or NEUROG3 signalling. NEUROG3, NKX6.1 and PDX1 expression was measured upon treatment. Each compound was assessed in 3–4 concentrations. Data are presented as mean with standard deviation. Gene expression analysis is presented as 2(-Delta Delta C(T)). n = 2 repeats on 3 hPDO lines.

SUPPLEMENTARY TABLE S3 Rho associated kinase inhibition reduces number of CHGA+ cells after NEUROG3 induction **(A)** Schematic overview of NEUROG3-hPDO differentiation upon treatment with the Rho associated kinase inhibitor Y27632. NEUROG3-hPDOs were harvested on day 7 of the differentiation for analysis. **(B)** Brightfield microscopy images of NEUROG3-hPDOs after 7 days of differentiation. hPDOs treated with Y27632 retained their cystic morphology when compared to the basal differentiation medium. **(C)** Quantification of CHGA+ cells by flow cytometry revealed a reduction in CHGA+ cells upon treatment with Y27632. Data are presented as mean with standard deviation. n = 2 repeats on 3 hPDO lines.

SUPPLEMENTARY VIDEO S1 NEUROG3-P2A-dTomato expression starts within 24 h of doxycycline induction Confocal live imaging of NEUROG3-hPDO after addition of doxycycline. Video presented as a Z-stack projection of a cross-section. NucRed™ Dead 647 ReadyProbes™ Reagent was added to the media during imaging to visualize viability. dTomato expression shown in red, and dying cells shown in green. NEUROG3 expression is approximately 7 h post-induction, with a peak 15-hours post-induction.

SUPPLEMENTARY VIDEO S2 NEUROG3-hPDOs adopt a condensed morphology after NEUROG3 expression is induced Confocal live imaging of NEUROG3-hPDO after addition of doxycycline. Video presented as a Z-stack projection of a cross-section. NucRed™ Dead 647 ReadyProbes™ Reagent was added to the media during imaging to visualize viability. dTomato expression shown in red, dying cells shown in green. After NEUROG3 induction, hPDOs adopt a less cystic morphology.

SUPPLEMENTARY VIDEO S3 Delamination of live cells from the NEUROG3-hPDO Confocal live imaging of NEUROG3-hPDO 24 h after addition of doxycycline. Video presented as a single cross-section. NucRed™ Dead 647 ReadyProbes™ Reagent was added to the media during imaging to visualize viability. dTomato expression shown in red, dying cells shown in green. Delamination of live cells can be detected 24 h after NEUROG3 induction, from a thickened epithelial lining of the hPDOs.

SUPPLEMENTARY VIDEO S4 Delamination 48 h after induction of NEUROG3 Confocal live imaging of NEUROG3-hPDO 24 h after addition of doxycycline. Video presented as a single cross-section. NucRed™ Dead 647 ReadyProbes™ Reagent was added to the media during imaging to visualize viability. dTomato expression shown in red, dying cells shown in green. Following delamination of live cells from hPDOs up to 48 h shows a less organized pattern. Cells rapidly exit the delamination site, often leaving behind gaps in the hPDO epithelial lining, likely due to a ubiquitous expression of NEUROG3 within the hPDOs.
