# Supplementary figures and images for: Effects of Neurogenin 3 Induction on Endocrine Differentiation and Delamination in Adult Human Pancreatic Ductal Organoids
Source: Transpl Int. 2025 Apr 1;38:13422. doi: 10.3389/ti.2025.13422 (PMC11996654; doi:10.3389/ti.2025.13422)

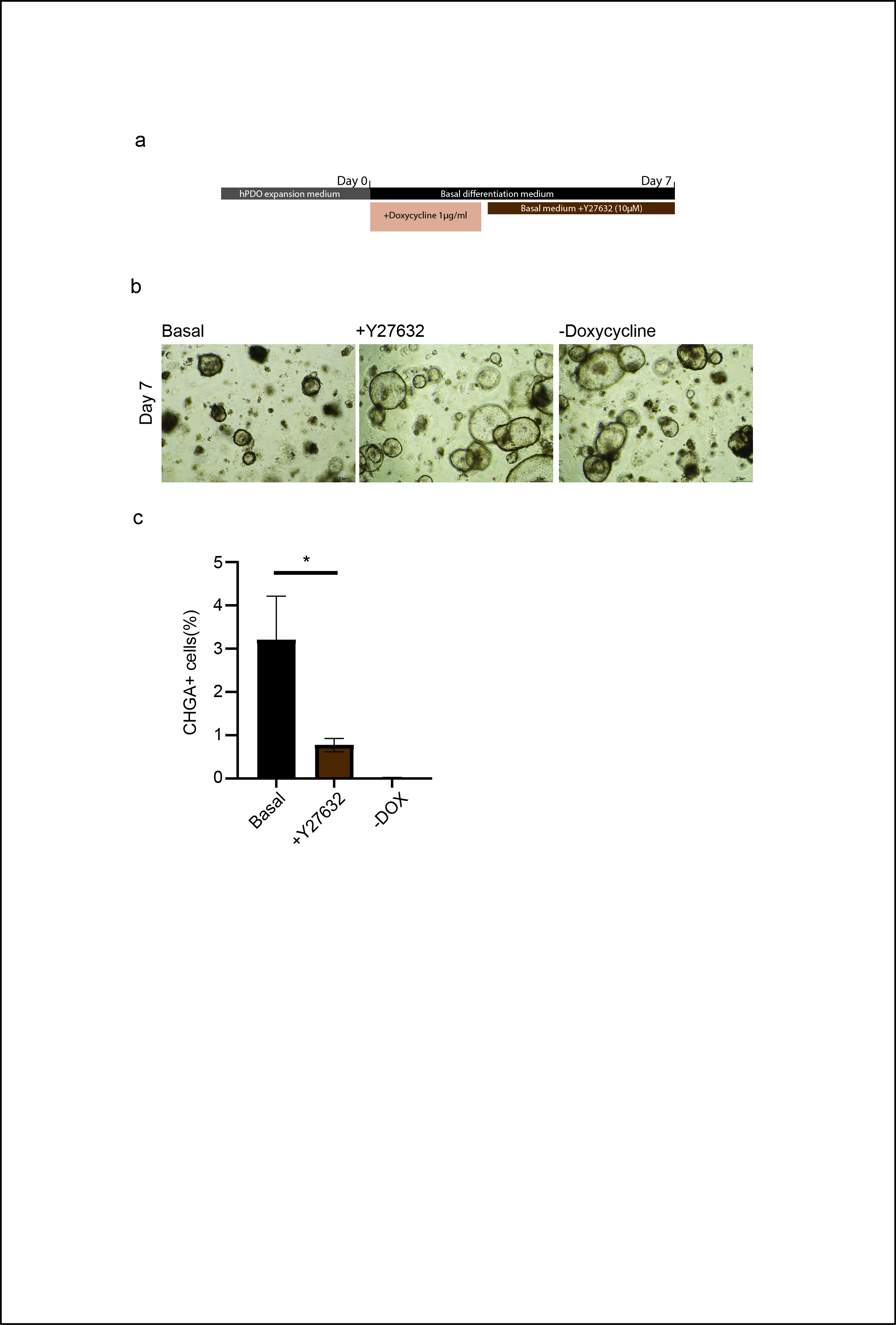

Supplement: Supplementary file 1 [file Image3.jpg]

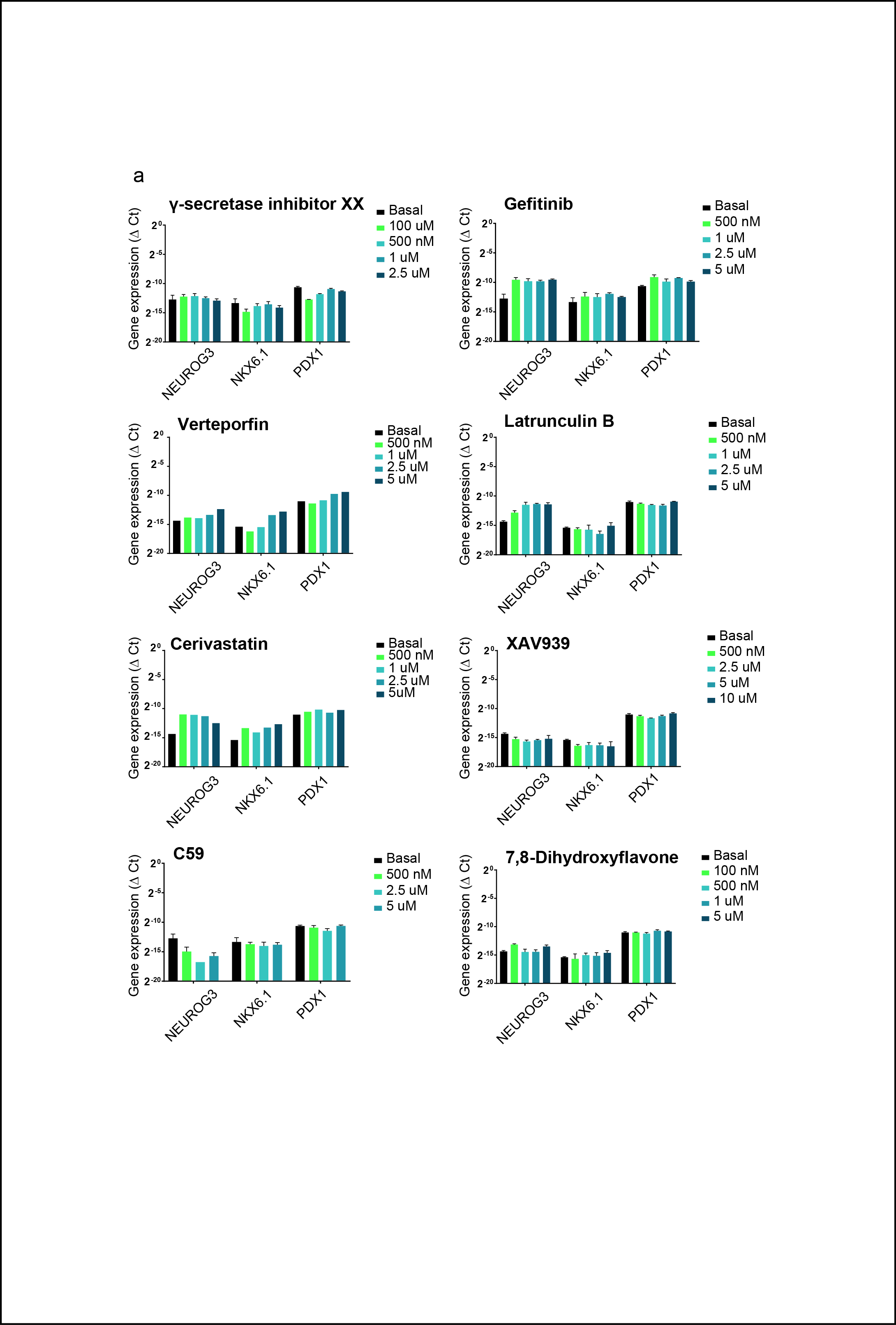

Supplement: Supplementary file 2 [file Image2.jpg]

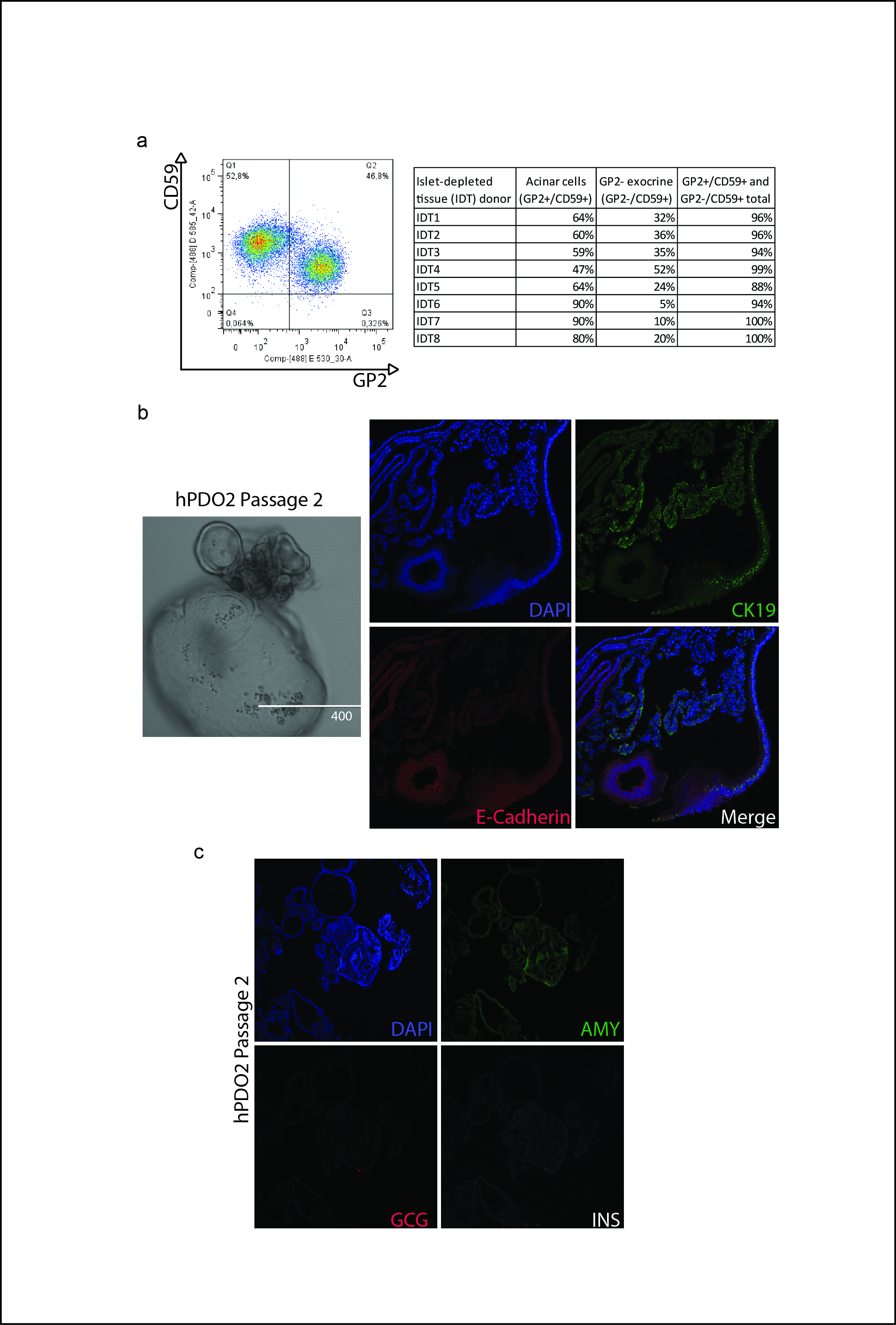

Supplement: Supplementary file 8 [file Image1.jpg]
