# Supplementary material for: Effects of Neurogenin 3 Induction on Endocrine Differentiation and Delamination in Adult Human Pancreatic Ductal Organoids
Source: Transpl Int. 2025 Apr 1;38:13422. doi: 10.3389/ti.2025.13422 (PMC11996654; doi:10.3389/ti.2025.13422)
Supplement: Supplementary file 3 [file Table1.docx]

Supplementary Table 1 – Organ donor characteristics

| **hPDO ID^*^** | **Sex** | **Age** | **BMI^**^** | **Cause of Death** |
| --- | --- | --- | --- | --- |
| hPDO1 | Male | 71 | 25 | Cardiac arrest |
| hPDO2 | Male | 54 | 23 | Subdural hematoma |
| hPDO3 | Male | 68 | 29 | Euthanasia |

* hPDO = human pancreatic ductal organoid

** BMI = Body Mass Index
