# Supplementary material for: Effects of Neurogenin 3 Induction on Endocrine Differentiation and Delamination in Adult Human Pancreatic Ductal Organoids
Source: Transpl Int. 2025 Apr 1;38:13422. doi: 10.3389/ti.2025.13422 (PMC11996654; doi:10.3389/ti.2025.13422)
Supplement: Supplementary file 5 [file Table2.docx]

Supplementary Table 2 – Compounds used in differentiation experiments

| Compound | Final concentration | Catalogue number | Company |
| --- | --- | --- | --- |
| ALK5 inhibitor II (ALK5i2) | 10 µM | sc-221234B | SCBT |
| Cerivastatin (CER) | 0.5 µM | SML0005 | Sigma-Aldrich |
| Wnt-C59 (C59) | 0.5 µM | 2287 | Axon Medchem |
| Gefitinib (GEF) | 2.5 µM | 1393 | Axon Medchem |
| Gamma secretase inhibitor XX (γ-s XX) | 5 µM | SML0649 | Sigma-Aldrich |
| Latrunculin B (LatB) | 1 µM | 428020 | Sigma-Aldrich |
| PD0325901 (PD032) | 100 nM | 1408 | Axon Medchem |
| R428 | 2 µM | 1946 | Axon Medchem |
| ROCK-inhibitor (Y-27632) | 10 µM | 1683 | Axon Medchem |
| Thyroid hormone (T3) | 1 µM | T6397 | Sigma-Aldrich |
| Protein kinase C activator TPPB (TPB) | 100 nM | 565740 | EMD Millipore |
