# Supplementary material for: Effects of Neurogenin 3 Induction on Endocrine Differentiation and Delamination in Adult Human Pancreatic Ductal Organoids
Source: Transpl Int. 2025 Apr 1;38:13422. doi: 10.3389/ti.2025.13422 (PMC11996654; doi:10.3389/ti.2025.13422)
Supplement: Supplementary file 6 [file Table3.docx]

Supplementary Table 3 – Primers used in qRT-PCR analysis

| Gene | Forward primer | Reverse primer |
| --- | --- | --- |
| CHGA | AAGGGGATACCGAGGTGATG | GTTTCTTCTGCTGATGTGCCC |
| GADPH | TCAAGAAGGTGGTGAAGCAGG | ACCAGGAAATGAGCTTGACAAA |
| GCG | GCATTTACTTTGTGGCTGGA | CGCTTGTCCTCGTTCATCT |
| INS | AGGCCATCAAGCAGATCACT | TTCCCCGCACACTAGGTAGA |
| ISL1 | TCGCCTTGCAGAGTGACATA | TCGCCTTGCAGAGTGACATA |
| MAFA | TTCTCCTTGTACAGGTCCCG | GAGAGCGAGAAGTGCCAACT |
| NEUROD1 | TGAGACTATCACTGCTCAGG | CACTCTCGCTGTACGATTTG |
| NGN3 | CGCTGCTCATCGCTCTCTA | CTCCGTCTCACGGGTCAC |
| NKX2.2 | CCTTCTACGACAGCAGCGACA | AGACCGTGCAGGGAGTACTGA |
| NKX6.1 | CACGAGACCCACTTTTTCCG | TCCCCAACGAATAGGCCAAA |
| PAX4 | AACAGCACCAGAAAGGTGTC | CAAAGCAGTCCTGAGTCCAG |
| PDX1 | CCATGGATGAAGTCTACCAAAGCT | CGTGAGATGTACTTGTTGAATAGGAACT |
| RPLP0 | GGCGTCCTCGTGGAAGTGAC | GCCTTGCGCATCATGGTGTT |
| SNAI2 | ACACATACAGTGATTATTTCCCCGT | AGGAGAGAGGCCATTGGGTA |
| SST | CCCAGACTCCGTCAGTTTCT | ATCATTCTCCGTCTGGTTGG |
