# Supplementary material for: Effects of Neurogenin 3 Induction on Endocrine Differentiation and Delamination in Adult Human Pancreatic Ductal Organoids
Source: Transpl Int. 2025 Apr 1;38:13422. doi: 10.3389/ti.2025.13422 (PMC11996654; doi:10.3389/ti.2025.13422)
Supplement: Supplementary file 7 [file Table4.docx]

Supplementary Table 4 – List of antibodies

| Antibody | Supplier | Identifier | Origin | Dilution |
| --- | --- | --- | --- | --- |
| Chromogranin A | Abcam | Cat# ab15160;  RRID:AB_301704 | Rabbit | 1:500 |
| Nkx6.1 Alexa Fluor® 647 | BD Biosciences | Cat# 563338;  RRID:AB_2738144 | Mouse | 1:200 |
| Alpha-Amylase | Cell Signaling Technology | Cat# 3796;  RRID:AB_2226822 | Rabbit | 1:200 |
| Cytokeratin 19 | Abcam | Cat# ab15463;  RRID: AB_2281021 | Rabbit | 1:50 |
| E-Cadherin | BD Biosciences | Cat# 610182;  RRID:AB_397581 | Mouse | 1:1000 |
| Glucagon | Abcam | Cat# ab10988;  RRID: AB_297642 | Mouse | 1:200 |
| Insulin | Abcam | Cat# ab7842;  RRID: AB_306130 | Guinea Pig | 1:100 |
| Anti-rabbit IgG Alexa Fluor 488 | Invitrogen | Cat# A-11008;  RRID: AB_143165 | Goat | 1:2000 |
| Anti-Mouse IgG Alexa Fluor 568 | Invitrogen | Cat# A-11004;  RRID: AB_2534072 | Goat | 1:1000 |
| Anti-Guinea Pig IgG Alexa Fluor 647 | Invitrogen | Cat# A-21450;  RRID: AB_141882 | Goat | 1:1000 |
